# A research capacity strengthening project for infectious diseases in Honduras: experience and lessons learned

**DOI:** 10.3402/gha.v6i0.21643

**Published:** 2013-08-07

**Authors:** Ana Lourdes Sanchez, Maritza Canales, Lourdes Enriquez, Maria Elena Bottazzi, Ada Argentina Zelaya, Vilma Esther Espinoza, Gustavo Adolfo Fontecha

**Affiliations:** 1 Department of Community Health Sciences, Brock University, St. Catharines, Ontario, Canada; 2 Escuela de Microbiología, Universidad Nacional Autónoma de Honduras (UNAH), Tegucigalpa, Honduras; 3 Instituto de Investigaciones en Microbiología, UNAH, Tegucigalpa, Honduras; 4 National School of Tropical Medicine, Baylor College of Medicine and Texas Children's Hospital, Houston, TX, USA; 5 Centro Nacional de Capacitación en Bioseguridad, UNAH, Tegucigalpa, Honduras

**Keywords:** research capacity strengthening, infectious disease, global health, Millennium Development Goals, Honduras

## Abstract

**Background:**

In Honduras, research capacity strengthening (RCS) has not received sufficient attention, but an increase in research competencies would enable local scientists to advance knowledge and contribute to national priorities, including the Millennium Development Goals (MDGs).

**Objective:**

This project aimed at strengthening research capacity in infectious diseases in Honduras, focusing on the School of Microbiology of the National Autonomous University of Honduras (UNAH). The primary objective was the creation of a research-based graduate program for the continued training of researchers. Parallel objectives included institutional strengthening and the facilitation of partnerships and networks.

**Methods:**

Based on a multi-stakeholder consultation, an RCS workplan was designed and undertaken from 2007 to 2012. Due to unexpected adverse circumstances, the first 2 years were heavily dedicated to implementing the project's flagship, an MSc program in infectious and zoonotic diseases (MEIZ). In addition, infrastructure improvements and demand-driven continuing education opportunities were facilitated; biosafety and research ethics knowledge and practices were enhanced, and networks fostering collaborative work were created or expanded.

**Results:**

The project coincided with the peak of UNAH's radical administrative reform and an unprecedented constitutional crisis. Challenges notwithstanding, in September 2009, MEIZ admitted the first cohort of students, all of whom undertook MDG-related projects graduating successfully by 2012. Importantly, MEIZ has been helpful in expanding the School of Microbiology's traditional etiology-based, disciplinary model to infectious disease teaching and research. By fulfilling its objectives, the project contributed to a stronger research culture upholding safety and ethical values at the university.

**Conclusions:**

The resources and strategic vision afforded by the project enhanced UNAH's overall research capacity and its potential contribution to the MDGs. Furthermore, increased research activity and the ensuing improvement in performance indicators at the prime Honduran research institution invoke the need for a national research system in Honduras.

Honduras is categorized as a medium-development country, ranking 120 among 187 countries and territories globally (1). It is located in Central America's extreme north, within the Mesoamerica Biodiversity Hotspot (2). It is a relatively small country (112,492 km^2^) with a population of over 8 million inhabitants (3). Within the Americas, Honduras is considered one of the least developed nations (4), characterized by profound social inequalities (5). While important progress has been made toward achieving the Millennium Development Goals (MDGs), serious challenges remain as the country not only endures the double burden of non-communicable and infectious diseases (3) but also a number of social issues dominating the domestic political agenda (6). Perhaps, not surprisingly, despite the global movement for strengthening health research as an essential factor for human development (7, 8), Honduras has yet to prioritize scientific research (9). Currently, the country is considered among the least scientifically developed in Latin America (10) and the world (11, 12).

However, institutional and individual efforts to increase Honduras's research productivity are ongoing. The National Autonomous University of Honduras (UNAH) is the prime Honduran higher education institution (13, 14) but as is often the case for developing countries’ universities (15, 16), UNAH's strength resides in undergraduate teaching, while scientific research is limited (only 3% of >3,000 faculty members are registered as researchers) (13). Researchers at UNAH are concentrated in a few academic units, especially at the School of Microbiology (13). Reasons for this particularity include, first, that most faculty members hold graduate degrees (mainly MSc) earned abroad; and second, that the School was one of UNAH's main beneficiaries of a Swedish initiative to increase regional research capacity in medical microbiology (14, 17, 18). From 1988 to 2012, the Swedish cooperation through the Karolinska Institute Research Training (KIRT) program trained 11 Honduran microbiologists. Regrettably, only five of them remain at UNAH, while the rest moved to Sweden, or took non-leadership positions elsewhere. In addition to facing the widely known constraints foreign-trained scientists encounter upon returning to their native developing countries (19), Honduran KIRT graduates went back to an academic unit lacking a graduate program where to consolidate their scientific capacity. As a consequence, their research activity gradually declined. In contrast, most of their Costa Rican counterparts returning to their homeland – with a strong research tradition (11, 20) and one of the most integrated NHRS in Latin America (8, 20, 21) – enrolled into active research laboratories affiliated with graduate schools, and continued to do research and produce peer-reviewed publications (22, 23). Honduras's case is not unusual but suggests that the concept of research capacity strengthening (RCS) characterizing donors’ initiatives for decades (24) needs to be advanced. Nowadays, a consensus exists that a long-term, systemic, and inter-sectoral approach is necessary to sustain an active local scientific community (19, 25). Arguably, centers of excellence and permanent graduate programs are at the core of thriving research environment (26, 27).

This article describes the implementation, challenges, and lessons learned of an RCS project entitled ‘Increasing Capacity to Achieve MDG No. 6 in Honduras: Combating Infectious Diseases’ (http://www.brocku.ca/globalhealth). The primary objective was establishing an infectious disease graduate program that would serve as permanent platform for continued scientific research at UNAH's School of Microbiology.

## Project conceptualization

Responding to a request for applications (RFA) issued in 2006 by the Teasdale-Corti Team Grants Program of the Global Health Research Initiative (GHRI), the core working team and authors of this article submitted a proposal for this project. The RFA entailed a proposal development grant (PDG) and a full proposal application. The first three authors of this article learned of this opportunity while working on a related Honduras-based project. The core team assembled and submitted a PDG proposing the creation of a graduate program, a long-standing goal at the School of Microbiology. Initial PDG funds were granted to conduct a national consultation to discuss the need and feasibility of such a program. The consultation took place in 2006, gathering in a two-day workshop a wide range of stakeholders: practitioners, educators, researchers, and research users from infectious disease and related disciplines (microbiology, medicine, nursing, veterinary medicine, food and agriculture, public health, epidemiology, medical anthropology, sociology, education, non-governmental organizations, and funding agencies). Stakeholders identified three main barriers to infectious disease control in the country, namely, insufficient integration of disease-specific programs, limited expertise for program evaluation, and lack of highly trained professionals able to assist with control programs, do research, and advocate for evidence-based solutions. For the latter, a Honduras-based graduate program whose curriculum integrated the study of biomedical as well as social determinants of infectious disease was recommended. Research competencies identified in the literature as essential (28, 29) such as the ability to work collaboratively, efficiently, and ethically, as well as leadership and communication skills were indicated as strong assets in these future professionals. In addition to individual training, stakeholders highlighted the need for strengthening governance within academic and non-academic institutions. These recommendations were captured in a written declaration signed by all participants at the end of the workshop.

With all these elements, a full proposal was submitted outlining a 4-year program of work whose central objective was implementing a research-based, 2-year graduate program, named ‘the MSc Program in Infectious and Zoonotic Diseases’ (MEIZ, for its Spanish abbreviation). Of 259 PDGs and 35 full proposals received by GHRI, this project was one of 14 selected for funding (30).

## Objectives

Our work focused on the following objectives:

### Objective 1

To create a sustainable research-based graduate program that integrates the study of Honduran-relevant biomedical, social, and environmental drivers of infectious diseases, including zoonoses.

### Objective 2

To strengthen research capacity at UNAH, focusing on research methodology, while promoting better practices and policies around research ethics and biosafety.

### Objective 3

To foster a favorable research environment that provides UNAH's research community the essential resources to generate and disseminate research.

### Objective 4

To promote the development and strengthening of networks so Honduran investigators can form or maintain partnerships at the national, regional, and international levels.

## Methods

The project was conceptualized as ‘capacity-strengthening’ in contrast to ‘capacity-building’. Both terms are often regarded as synonymous but we coincide with other authors that the former is more encompassing as it conveys the intention to enhance pre-existing capacity (31). Our project implementation used a ‘multi-level approach’ (32). Ideally, RCS interventions should consider all levels (individual, institutional, and if possible, the macro-level context), as each is affected by the other (33). The present project, while focusing on individuals, also endeavored to promoting networks from which individuals could influence the system. It also strived for facilitating an enabling environment at the institutional level. This approach is considered an advanced model of capacity building (28) and increases the likelihood of sustainability (27). Whitworth and co-workers propose a similar approach, but explicitly recommend the ‘engagement of southern voices and institutions’ (34). This engagement was also an integral part of the present project.

Pedagogy specialists at UNAH provided advice to design and implement the graduate program as outlined in the consultation workshop. They recommended a plan comprising modules on the following: Honduran-specific content (epidemiology of infectious disease, health system, national plan, and so on); a primer on global health; discipline-specific content (e.g. pathogens, immunology, diagnostics); and experiential learning opportunities through field visits and laboratory sessions. Cross-cutting competencies, such as research methodology, biosafety, research ethics, technical writing, and project management, were integrated through periodic activities. Research seminars took place throughout the program, and an original research project was a requisite for degree completion.

Other theoretical foundations helpful for project implementation are as follows. Change theory and social cognitive (learning) theory (35) helped us implement self-efficacy strengthening activities for students. Individual mentoring along with group seminars and skill building laboratories resulted in increased academic performance and motivation. Organizational change theory (36) helped keep at reasonable levels the team's expectations of influencing sustainable organizational change within UNAH. According to Buchanan et al., sustainability of change within an organization is a complex phenomenon affected by internal factors as much as by a number of externalities outside an individual project's control (36). Social capital and networks theory (37) informed the formulation of a specific objective for promoting networks as means of preserving and consolidating a critical mass of researchers.

Finally, to address the power differentials operating in North–South partnerships (17, 38, 39) we strived for a truly cooperative partnership based on trust and mutual respect. According to Costello and Zumla, this type of partnership rests on four principles: (1) mutual trust and shared decision making; (2) national ownership; (3) emphasis on getting research findings into policy and practice; and (4) development of national research capacity (38).

### Monitoring and evaluation

Internal monitoring and evaluation (M&E) activities were ongoing. Logic framework approach (LFA) was used initially and we periodically measured objectives’ achievements using a quantitative scale. But setbacks experienced during the first 2 years made it impossible to launch MEIZ. Hence, our midterm evaluation showed that the project was failing. These raised concerns among project partners including the funding agency, which encouraged us to reconsider our objectives and/or find a different Honduran partner. However, upon critical examination we decided that: (1) the project objectives were as relevant and valid as ever; (2) we have made great strides toward achieving those objectives; and (3) we were strengthening capacity and promoting change in the process. Our M&E framework could not reflect this. After expert advice, we adopted Outcome Mapping (OM) (40), a process-oriented methodology that allows reporting incremental progress rather than just end-of-process outputs (40). Subsequent evaluations integrated both methodologies, so while we struggled accomplishing the original objectives, were able to show progress that otherwise would have gone unmentioned. This was useful for keeping the team's morale and the Canadian partners’ confidence.

## Activities and experience

For our first objective (MEIZ creation and implementation), we carried out a plan involving the following stages: curriculum design (as described above), establishing administrative and academic infrastructures, implementation, internal assessment, accreditation preparedness, and planning for sustainability.

Nine students were admitted to MEIZ. Due to the full-time nature of the program, tuition scholarships and modest research fellowships were offered. Regardless, requiring exclusive dedication to the program limited the number of applicants to only those who could afford not holding a full-time job or those whose employers were able to sponsor their studies.

Two additional points are worth emphasizing. First, in the absence of graduate handbooks or similar academic guidelines at UNAH, we implemented an adapted a version of the MSc graduate handbook in use at Brock University's Faculty of Applied Health Sciences. Briefly, in addition to the supervisor, an advisory committee was appointed for each student to facilitate his or her progress. Beginning the second year, students had to defend their project's proposal; a process that entailed completing the thesis’ first three chapters (introduction, literature review, and methodology) and a short oral defence. Upon the project's completion, students had to undergo a formal defence for which an external examiner was required. At this point, a complete thesis document (including results, discussion, conclusions, and recommendations) was mandatory.

The second point deserving mention is about sustainability. To avoid problems experienced by other programs operating unsanctioned by the Higher Education National Council, we were determined not to launch MEIZ without proper approvals. This caused a 1-year delay, but it assured MEIZ's continuation. Academic sustainability was secured by strengthening faculty members’ methodological and supervisory skills and providing them with multiple opportunities for professional development. Implementing a biosafety level-2 research laboratory, installing a research ethics board (REB), and prioritizing biosafety practices enhanced the research environment. Activities related to Objectives 2–4 are summarized in Tables 1–3. Briefly, to contribute to institutional strengthening we conducted needs assessments on four domains (methodology, graduate studies, research ethics, and biosafety) (41) and implemented responsive work plans including continuing education, acquisition of additional funding, expert consultation, and so on.

**Table 1:** Institutional strengthening activities undertaken at National Autonomous University of Honduras (UNAH) and the School of Microbiology

Domains	Actions taken by the project
**Methodology**	
Weaknesses in	
Research design Literature search Knowledge synthesis Statistical analysis Scientific writing Results dissemination Project management Project monitoring and evaluation	Established a ‘train-the-trainers’ program Organized and delivered courses, workshops, and hands-on training experiences Facilitated/funded attendance to conferences and trainings Increased library collection Gave access to bibliographic material and software Implemented individual and group mentoring
**Graduate studies**	
Lack of a research-based graduate program in infectious diseases Disconnect between research and graduate studies offices Low visibility of graduate programs	Designed, created, and implemented MEIZ Created dialog opportunities Facilitated participation at UNAH's research activities and publications Promoted research through mass communication media Promoted MEIZ at different scientific and policy-making venues
**Biosafety**	
Low awareness of the importance of biosafety practices Inadequate expertise Irregular use of biosafety precautions Lack of internal biosafety guidelines Minimally active Biosafety Committee Inadequate supplies and signage Absence of continuing education on biosafety	Integration of biosafety into undergrad/grad curricula Facilitated courses, seminars, and onsite visits by international experts Increased library collection Biosafety assessment by an international expert Training and courses for students, faculty, and staff by local and international instructors Reactivation of Biosafety Committee Appointment of a biosafety officer for MEIZ Supplied personal protective equipment and printed resources Created the National Center for Biosafety Training (CENCAB)
**Research ethics**	
Low awareness of the need for research ethics clearance for research with human participants Inadequate expertise Absence of institutional research ethics board Lack of guidelines for research ethics oversight Absence of continuing education on research ethics	Integrated research ethics into undergraduate and graduate curricula Provided access to online training (www.citiprogram.org) Facilitated courses, seminars, and hands-on training Facilitated and funded onsite visits by experts Increased library collection Appointment of an ethics officer for MEIZ and School Drafted guidelines, protocols, and standard operating procedures for research ethics review process Implemented a research ethics board for MEIZ Obtained additional funding through the GHRI's Global Health Research Awards (GHLA) initiative (see GHLA's website at www.brocku.ca/globalhealth/ghla.php) Created the Documentation Centre for Bioethics and Research Ethics (C-BIO)

MEIZ: Master's Program in Infectious and Zoonotic Diseases.

**Table 2:** Spaces allocated by the National Autonomous University of Honduras (UNAH) to the Honduras-Canada Teasdale-Corti project

Physical/intellectual space	Project's contribution	Location	Current usage
MEIZ classroom	Design, renovations, and furnishings	School of Microbiology Building J-1, 4th Floor	MEIZ School of Microbiology lectures and seminars Research ethics board
MEIZ administrative office	Design, renovations, and furnishings	School of Microbiology Building J-1, 4th Floor	MEIZ Office space for projects’ PIs
Teasdale-Corti Research Lab	Design, renovations, equipment, and laboratory furniture	School of Microbiology Building J1, 4th Floor	MEIZ graduate students Faculty members associated with project Other researchers at the School of Microbiology and UNAH
Conference room	Furnishing	Sciences building Building E-1, 2nd Floor	MEIZ Microbiology lectures and seminars Other academic units
CENCAB			
National Center for Biosafety Training Opened on April 2012	Concept and design Consultationfurnishing, signage	Sciences building Building E-1, 2nd Floor	Biosafety training for UNAH's health/biosciences and chemical engineering students, faculty, and custodial staff. Others from private and public sector
C-BIO Documentation Centre for Bioethics and Research Ethics Inaugurated on August 1, 2012	Concept and design furnishings, library collection	UNAH's central library	University-wide access Research ethics boards General public

MEIZ: Masters Program in Infectious and Zoonotic Diseases.

**Table 3:** Alliances and networks facilitated by the Honduras-Canada Teasdale-Corti project

Institution	Period	Activity or project
**National**		
National University of Agriculture (UNA)	2007–2012	Training MEIZ students Research projects Community involvement
Ministry of Health (MoH)	2007–2012	MoH staff admitted to MEIZ Biosafety seminars Collaboration with research projects on malaria, dengue, and soil-transmitted helminths Part of the steering committees for (a) The National Plan for Neglected Diseases (b) Malaria (‘Mesa Técnica’) (c) NHRS
PAHO-Honduras	2011–2012	Research ethics initiative Advisory Committee on Health Research of the Pan American Health Organization (ACHR)
**International**		
Brock University, Canada	2006–2012	Overall project leadership Financial stewardship Ethics and biosafety expertise Pedagogical and methodological expertise Graduate program design and direction Joint conference presentations Peer-reviewed publications Alliances brokerage
George Washington University, USA	2007–2011	Project evaluation Collaboration with research projects Supervision of graduate students Joint conference presentations
Baylor College of Medicine, USA	2011–2012	Supervision of graduate students Network facilitation Joint conference presentations
Emory University, USA	2008–2010	Biosafety assessment and training Joint publications and conference presentations NeTropica Meeting
Centers for Disease Control, USA	2011	Malaria training
Mexican Biosafety Association	2011–2012	Biosafety training and expertise. Expert support to CENCAB
Canadian Coalition for Global Health Research, CCGHR	2010–2012	Global health advice Networking NeTropica meeting
University of Guelph	2011	Research on healthcare access in Honduras
McMaster University, Canada	2009–2012	Dengue genetics project MEIZ thesis co-supervision
University of Calgary	2009–2010	Workshop on Zoonotic diseases
Sanger Institute, UK	2009–2012	Co-supervision of MEIZ student Joint conference presentations NeTropica Meeting
NeTropica, Costa Rica	2007–2012	Funding MEIZ students NeTropica Meetings Alliances brokerage
University of Costa Rica	2009–2012	Training and supervising MEIZ students Joint conference presentations
National University of Costa Rica	2011–2012	Entomology training for MEIZ students
University of San Carlos, Guatemala	2008–2010	Research Ethics training and mentoring
National University of Nicaragua (UNAN) Leon, Nicaragua	2007–2012	Curricular design, MEIZ seminars, and students training
TDR	2009–2011	Membership in the Disease Reference Group on Zoonoses and Marginalised Infectious Diseases (DRG6)
COHRED	2012	NeTropica Meeting, networking with Council of Ministers of Health from Central America and Dominican Republic (COMISCA)

COHRED: Council on Health Research for Development; GHRI: Global Health Research Initiative, Canada; MEIZ: Masters Program in Infectious and Zoonotic Diseases; NeTropica: The Network for Research and Training in Tropical Diseases in Central America; NHRS: National health research system; TDR: Special Programme for Research and Training in Tropical Diseases of the World Health Organization.

To foster a favorable research environment, we secured and furnished research spaces, as shown in Table 2. We also organized a variety of courses and conferences, facilitated attendance by our associates to scholarly meetings, and promoted networks and partnerships regionally and internationally (Table 3).

## Outputs

### RCS outputs

#### Graduate students

All nine students completed the program, for an unprecedented 100% graduation efficiency at UNAH. Project topics and supervisors’ affiliation are listed in Table 4.

**Table 4:** Research project topics and supervisors’ affiliation for MEIZ graduates first cohort

Research project topic	Supervisors’ affiliations
Genetic characterization of *Chlamydia trachomatis*	Primary: Baylor College of Medicine, USA Co-Supervisor: Sanger Institute, UK
	
Genetic characterization and drug resistance of Methicillin-resistant *Staphylococcus aureus* (MRSA)	Primary: University of Costa Rica Co-supervisor: UNAH
	
Molecular epidemiology of enteric viruses causing diarrheal disease in children under 5 years of age	Primary: UNAH Co-supervisor: UNAH
	
Susceptibility of larval stages *of Aedes aegypti* to Temephos	Primary: McMaster University, Canada Co-supervisor: UNAH
	
Genetic characterization of *Histoplasma capsulatum* isolated from Honduran patients	Primary: Brock University, Canada Co-supervisor: UNAH
Interactions of *Brucella canis* with eukaryotic cells	Primary: University of Costa Rica Co-supervisor: UNAH
	
Human host genetics and severity of Dengue infections	Primary: UNAH Co-supervisor: University, Leon, Nicaragua
	
Bacterial etiology of diarrhea in children under 5 years of age	Primary: UNAH Co-supervisor: UNAH
	
Soil-transmitted helminth infections in Honduran school children	Primary: Brock University, Canada Co-supervisor: UNAH

UNAH: National Autonomous University of Honduras; MEIZ: Master's Program in Infectious and Zoonotic Diseases.

#### Teaching resources

We implemented the ‘School of Microbiology Advancement Grants’ to support undergraduate teaching and strengthen grantmanship skills. Projects funded included an online microbiology magazine, writing laboratory manuals, expanding bacterial collections, training in molecular techniques, quality control in haematology, and optimization of immune assays.

#### Infrastructure

We transformed or supported a number of physical spaces for teaching and research. Due to their impact, the following three are worth mentioning:

1. The Teasdale-Corti Research Laboratory, a fully equipped biosafety level-2 facility accessible to researchers and students across the university;
2. The Documentation Center for Bioethics and Research Ethics (C-BIO) a space designed to evoke reflection about ethics, academic integrity, and respect for the environment; and
3. The National Center for Biosafety Training (CENCAB), the first and only in Honduras, offering services to the private and public sectors. To date CENCAB had trained UNAH's custodial services and laboratory personnel as well as > 2,000 students.

#### Networks and partnerships

Project activities fostered frequent interactions between Honduran researchers with regional, North American, and European researchers (see Table 3). To consolidate MEIZ position in Central America, we hosted the VI Biennial NeTropica Meeting, held in Copán, Honduras, July 2012. Several collaborations forged at the meeting are already taking place (more about this meeting at: http://www.brocku.ca/globalhealth/nett2012.php). NeTropica (Network for Research and Training in Tropical Diseases in Central America, http://www.netropica.org) was created with Swedish funding to help KIRT graduates establishing a regional scientific community in the field of tropical diseases (18).

### Research outputs

#### Reports

We delivered 6 reports to UNAH and 19 technical research reports to the funding agency (11 progress reports, 1 midterm evaluation, 1 extension request, 5 annual reports, and 1 final report).

#### Conference presentations

Collectively, we made a total of 48 international conference presentations, 50% of which were student-driven.

#### Publications

MEIZ students have published in several journals, for example, in UNAH's research journal (*n*=3), the Honduran Medical Journal (*n*=1), a Costa Rican journal (*n*=1), and two international journals (*n*=2). Project team members have published eight peer-reviewed articles, one book chapter, and one biosafety manual.

#### Research studies

We also supported 15 collaborative investigations that either overlapped with MEIZ or were of interest to project associates. Projects ranged from graduate education and ethics, to food/water microbiology, malaria, soil-transmitted helminths, and zoonotic diseases.

### Policy and practice outputs

We helped establishing UNAH's first non-medical REB and making biosafety training compulsory for students exposed to biological and chemical hazards. We also assisted in revitalizing UNAH's Microbiology Research Institute and forged its alliance with MEIZ. Project team members have been appointed to leadership positions at CENCAB, MEIZ-REB, and the Research Institute; while others serve on national committees such as the National Program for Neglected Diseases, Malaria Task Force, and the Inter-Institutional NHRS Steering Committee. Project team members have also participated in international policy meetings; notably, the 2nd Latin American Conference on Research and Innovation for Health, the Global Forum for Health Research (a Geneva-based NGO committed to research and innovation for health, http://www.globalforumhealth.org/), and the Disease Reference Group on Zoonoses and Marginalised Infectious Diseases (DRG6) (42) convened in 2009 by the WHO-based Special Programme for Research and Training in Tropical Diseases (TDR).

## Outcomes

The project contributed to important changes in individuals as well as in the institution. First, we helped develop a Honduras-based graduate program reliant on local talent. In the past, UNAH had been the subject of different RCS models (from training opportunities in high-income countries to the ‘sandwich model’ implemented by the KIRT program) (17, 18). But we contended that although highly beneficial, such models left to chance the research environment those foreign-trained researchers would encounter upon returning to their home institutions. We also argued that a locally owned graduate program would not only boost research activity but also provide opportunities to those for whom foreign training is not a viable alternative.

Second, through MEIZ we succeeded in reinforcing a research culture that upholds scientific rigor as well as safety and ethical values. Third, since MEIZ integrates the study of biomedical and social determinants of infectious diseases, the program has been helpful in expanding the School of Microbiology's traditional etiology-based, disciplinary model to infectious disease teaching and research (43). Fourth, through mentoring sessions, we made every effort to demystify research, stimulate higher order thinking, and promote self-efficacy among students. Through OM exercises (40) students were able to set their own progress indicators, track behavior changes, and identify barriers to their learning.

Finally, the increased number of research partners engaged by the project, opened many opportunities for collaboration, mentoring, and behavior modeling. The project promoted multi-stakeholder meetings and brought together constituencies that normally have few opportunities to intersect. These linking opportunities are conducive to knowledge generation and innovation, and promote stronger research systems (17).

## Lessons learned

### The partnership

The fact that the Canadian principal investigator (PI) was a Honduran expatriate, former faculty member of the School of Microbiology and KIRT graduate, minimized the challenges that sometimes arise at the interpersonal level during RCS initiatives (31). Partnerships operating with expatriate research leaders can lead to sophisticated and yet neo-colonial models of collaborations (38), but the present partnership responded was relevant to the Honduran partner needs. The partnership, however, was not symmetrical as there were ‘inevitable constraints’ (39). The tasks requiring strong research proficiency fell heavily on the Canadian PI (e.g. grantmanship, technical writing, graduate program oversight, financial stewardship, research dissemination, networking). Consequently, the burden of responsibilities was, at times, daunting for the Canadian PI. As individual and institutional research capacities build up, more equitable distribution of responsibilities will be possible.

### The context

Although we originally planned this as a 4-year project, we were granted a no-cost 1-year extension. We experienced institutional-level challenges inherent to RCS initiatives (31) and also two unusual circumstances that imposed extraordinary difficulties. First, the transformation process happening at UNAH provoked widespread discoordination, high turnover of senior administrators, and frequent strikes. Second, the country's constitutional crisis that peaked in 2009 with the dismissal of the President (44, 45). The latter could have had disastrous effects on the project as it effectively halted foreign cooperation, impeded travel, and generated serious setbacks for MEIZ implementation. Three reasons explain why we were able to launch MEIZ in September 2009: the resilience of the team – an attribute identified as key factor in others settings (46), the understanding attitude of the funding agency, and the fact that we had built local capacity during the first 2 years.

### Multidisciplinarity quest

MEIZ was conceived with a multi-disciplinary curriculum to introduce students to the broad spectrum of infectious disease determinants and to instil the benefits of collaborative work. It was relatively easy to do this at the theoretical level (courses, seminars, field visits, and so on), but it proved more difficult for the thesis projects. (Table 4 shows that these projects mostly addressed basic science questions.) According to MEIZ design, it was preferable having students working collaboratively on common issues (e.g. on Dengue, a major infectious disease in the country, have students informing each other's work whether it was on genetics, health promotion, vector biology, and so on). We also intended working in collaboration with two related graduate programs existing in Honduras (Public Health and Epidemiology), by cross-listing courses, holding multi-disciplinary seminars, sharing supervisors, and so on. Scheduling incompatibilities, and to some extent, the lack of experience working together were barriers to this collaboration.

Achieving multidisciplinarity is not without challenges (47), but in the particular case of infectious disease, a collaborative approach to research and intervention is imperative if we are to reduce their burden to human and animal populations (43). As MEIZ continues to mature and the critical mass of researchers grows, we anticipate a diversification of research topics and collaboration across disciplines.

### MEIZ academic process and productivity

MEIZ was the first program at UNAH to implement a graduate handbook detailing a rigorous academic process. The handbook was based on a Canadian model, but far from being an inappropriate transfer, its implementation assured students’ timely completion. UNAH's lax regulations in this regard have had a counterproductive effect and few students formally graduate – if ever. Gradually building students’ ability for scientific writing through both a skills and process approach (48) was a critical determinant for MEIZ completion rate.

The number of students admitted to the program was distant from our original expectations. MEIZ was planned for commencement in 2008 and receiving one cohort per year thereafter. But we graduated one cohort and initiated the selection process of another. In this case, the scarcity of experienced Honduras-based supervisors was the limiting factor. To compensate, supervisors from abroad were recruited (Table 4), but importantly, supervisors affiliated with Canadian or US universities are Honduran expatriates. This supports the argument that ‘brain circulation does not have to be a zero-sum game’ (49) and underscores the benefits of knowledge networks. In time, availability of experienced supervisors will increase and so will the program's intake capacity. The latter will also depend on the possibility to offer full scholarships as MEIZ is committed to giving access to capable students regardless their financial situation.

### Sustainability

The measure of a sound development initiative resides in its sustainability (50). Intuitively, we made great efforts to promote self-reliance as an elemental factor for sustainability, but as it often happens with RCS initiatives (51), we did not agreed upon a precise set of short and long-term indicators of sustained capacity. The continued operation of the graduate program is, at present, the most obvious indicator of sustainability. With the project finalized, a period of adaptation will follow, as sustainability is a dynamic process and not a simple linear unfreezing, change, and refreezing of changes effected by the project (36). Instead of expecting UNAH to begin providing all resources needed for MEIZ's expansion, we envision an increased pursuit of national and international research funds as well as more proactivity in attracting research partners.

We recommend RCS initiatives to integrate a sustainability working framework to monitor the permanency and growth of the key capacities they aim to develop. Although defining, practicing, and measuring research capacity have gained significant attention recently (24, 27–29, 31, 52), frameworks are still needed for a systematic and empirical approach to RCS.

## Conclusions

Honduras is a scientifically lagging country (11) and has been, with few exceptions, ignored by international research organizations as well as by researchers from high-income countries. Our work is evidence that this can and should change (53).

While it is not possible to establish a linear cause–effect between the work presented here and the eventual long-term changes that may occur at individual and institutional levels (40), we would like to propose that the project was successful in contributing in a unique way to Honduras's research capacity. A distinguishable feature of this project is the utilization of a multi-level approach to capacity building, that is, a capacity strengthening model away from traditional models imposing uncritical transfers of training, resources, or research paradigms (31, 54). As such, this project helped reducing to some extent the research ‘asymmetry’ that prevents productive research collaboration (55). It also contributed in a way – however small – to Honduras's preparedness to meets its national objectives, including the MDGs.

Our results reveal that there is much talent and capacity in Honduras to advance scientific research and that adequate and opportune support at the individual and institutional levels are essential at this critical moment. The creation and effective operation of an NHRS in the country will undoubtedly help increase the amount and quality of health research. In turn, high-quality research will potentially benefit Honduras's human development.
